# Occult hepatitis C infection identified in injection drug users with direct antiviral agents therapy and spontaneous resolution of hepatitis C virus infection

**DOI:** 10.1016/j.virusres.2023.199104

**Published:** 2023-04-02

**Authors:** Eliane Silva, Sara Marques, Bárbara Leal, Bernardo Canhão, João Madaleno, Adélia Simão, Armando Carvalho

**Affiliations:** a Research Center in Biodiversity and Genetic Resources (CIBIO/InBIO), University of Porto, Vairão, Portugal; b School of Medicine and Biomedical Sciences of the University of Porto (ICBAS-UP), Porto, Portugal; c Unit for Multidisciplinary Research in Biomedicine (UMIB) at 2School of Medicine and Biomedical Sciences of the University of Porto (ICBAS-UP), Porto, Portugal; d Laboratory for Integrative and Translational Research in Population Health (ITR), Porto, Portugal; e Faculty of Medicine, University of Coimbra, Coimbra, Portugal; f Coimbra Hospital and University Center (CHUC), Coimbra, Portugal

**Keywords:** OCI, HCV, Drug users, IDUs, Direct-acting antivirals, RBCs

## Abstract

**Background:**

Occult hepatitis C infection (OCI) is characterized by the detection of hepatitis C virus (HCV) RNA in hepatocytes and in peripheral blood mononuclear cells (PBMCs) without detection in serum. We aimed to evaluate OCI in drug and no drug users who achieved sustained virological response (SVR) after therapy with direct-acting antivirals (DAAs) and with HCV spontaneous resolution.

**Methods:**

Twenty-four patients in the AVP group (who achieved a SVR after DAAs therapy), 13 in the NAVP group (with HCV spontaneous resolution) and 7 HCV-RNA positive patients (CPP, control positive group) were included in the study. HCV/OCI-RNA was screened in serum and PBMCs samples of the patients by ddPCR for OCI patients’ identification. Plasma and red blood cells (RBCs) samples of the patients were also evaluated for HCV/OCI-RNA detection by ddPCR.

**Results:**

OCI was presented in injection drug users (IDUs) in the AVP (20.8%) and NAVP (23.1%) groups by ddPCR with a higher statistically significant percentage detected in RBCs samples of the patients in the AVP group comparatively to NAVP (*p*<0.01) and CPP (*p* < 0.05) groups.

**Conclusion:**

OCI was identified in IDUs patients of the AVP and NAVP groups by ddPCR. These results suggest that OCI patients in the AVP group might not be entirely cured, and that OCI patients in the NAVP group were not identified at clinical evaluation time when just serum samples were analysed. A higher percentage of HCV/OCI-RNA was detected in RBCs samples. Overall results recommends that HCV/OCI identification in patients with DAAs therapy and spontaneous resolution of HCV infection should be studied more accurately in future and in larger patient groups if possible. Additionally, suggest also PBMCs and RBCs samples as predictors for HCV/OCI diagnosis and management.

## Highlights

• Occult hepatitis C infection (OCI) identified in injection drug users (IDUs) with direct-acting antivirals (DAAs) therapy and HCV spontaneous resolution) by droplet digital PCR (ddPCR).
• OCI IDUs with DAAs therapy might not be entirely cured.
• OCI IDUs without DAAs therapy identified due to tested PBMCs samples.
• Hight percentage of HCV-RNA detected in red blood cells (RBCs).
• HCV/OCI identification with PBMCs and RBCs samples as a predictor.

## Abbreviations

OCI: Occult hepatitis C infection
HCV: hepatitis C virus
RNA: ribonucleic acid
PBMCs: peripheral blood mononuclear cells
IDUs: injection drug users
NIDUs: no injection drug users
DAAs: direct-acting antivirals
GP: glecaprevir/pibrentasvir
LED/SOF: ledipasvir/sofosbuvir
SOF/VEL: sofosbuvir/velpatasvir
ELB/GRZ: elbasvir/grazoprevir
SVR: sustained virological response
NDUs: no drug users
ddPCR: droplet digital PCR
RBCs: red blood cells
CHUC: Coimbra Hospital and University Center
HIV: human immunodeficiency virus
AVP: patients who achieved a SVR after DAAs therapy
NAVP: patients with HCV spontaneous resolution
CPP: control positive group
RT-PCR: real time PCR
RPMI: roswell park memorial institute
FBS: fetal bovine serum
SPSS: statistics package for social sciences

## 1 Introduction

Occult hepatitis C infection (OCI) is characterized by the detection of hepatitis C virus (HCV) ribonucleic acid (RNA) in hepatocytes and peripheral blood mononuclear cells (PBMCs) without detection in serum by conventional PCR assays (Castillo et al., 2004).

The current understanding of OCI and its clinical implications remain unclear, although, this infection has been described in seropositive and/or seronegative patients with chronic liver disease, coinfections or comorbidities (Austria and Wu, 2018; Hedayati-Moghaddam et al., 2021; Naghdi et al., 2017; Frias et al., 2019; Wroblewska et al., 2021). Data describing that OCI could be present in many individuals who have “cleared” the virus spontaneously from their serum was also previously described (Yousif et al., 2018; Wang et al., 2019). Moreover, OCI was described in population with risk factors, such as in subjects with tattoos, acupuncture and apparently in healthy population (Martinez-Rodriguez et al., 2018; Helaly et al., 2017). Furthermore, it was also previously described in injection drug users (IDUs), and being this population a high-risk group for blood-borne infectious diseases transmission, OCI positive individuals could lead to HCV transmission (Sheikh et al., 2019; Donyavi et al., 2019; Sugden et al., 2013; Enkelmann et al., 2020). The role of HCV positive in no injection drug users (NIDUs), such as individuals that inhales crack cocaine, powder cocaine, consumes methamphetamines or heroin, could lead to HCV transmission if the sharing of their used pipes, straws or tubing were blood-contaminated, and OCI was also previously described in this population (Scheinmann et al., 2007; Van den Berg et al., 2009; Schuch-Goi et al., 2017).

The therapy with direct-acting antivirals (DAAs) of chronic hepatitis C markedly improved sustained virological response (SVR) rates, although OCI has been described in several conditions and after DAAs therapy (Yousif et al., 2018; Wang et al., 2019; Elmasry et al., 2017; Mohamed et al., 2019; Mekky et al., 2019). Likewise, HCV-cure in subjects with HCV or OCI infection that presented SVR after DAAs therapy may not be entirely valid (Attar and Van Thiel, 2015; Lybeck et al., 2019). Studies reporting patients’ treatment failure after DAAs therapy that used e.g. glecaprevir/pibrentasvir (GP), ledipasvir/sofosbuvir (LED/SOF), sofosbuvir/velpatasvir (SOF/VEL) and elbasvir/grazoprevir (ELB/GRZ) antivirals were previously described (Attar and Van Thiel, 2015; Lybeck et al., 2019; Ghany et al., 2020; Zahid et al., 2022).

Recently we have presented preliminary data regarding OCI patients treated or not-treated with DAAs (Silva et al., 2022) and also their possibility of HCV/OCI transmission (Silva et al., 2023), and here we aimed to evaluate OCI in drug and non-drug users (NDUs), including patients who achieved SVR after DAAs therapy (AVP group) and with spontaneous resolution of HCV infection (NAVP group). Serum and PBMCs samples were screened for HCV/OCI-RNA detection by droplet digital PCR (ddPCR), as this is a ultra-sensitive technology for low RNA viral load detection (Frias et al., 2019; Silva et al., 2023). Additionally, plasma and red blood cells (RBCs) patients’ samples were also screened for HCV/OCI-RNA detection by ddPCR.

## 2 Materials and methods

### 2.1 Study groups

Patients with HCV infection from the Coimbra Hospital and University Center (CHUC), between 2019 and 2021, were included in this study. All patients were anti-HCV positive and tested negative for hepatitis B virus and human immunodeficiency virus (HIV). A total of 44 patients (IDUs, NIDUs and NDUs) were included and divided in the following study groups: AVP - 24 patients (22 IDUs and 2 NDUs) with 8 or 12 weeks DAAs therapy who achieved an SVR-12; NAVP - 13 patients (4 IDUs, 6 NIDUs and 3 NDUs) with HCV spontaneous resolution. An HCV positive control group (CPP) (HCV RNA detected by real time PCR (RT-PCR)), including 7 NIDUs patients was also considered. Key clinicopathological data of these patients were collected and summed up in Table 1. Patients with confirmed HCV infection were treated with GP, LED/SOF, SOF/VEL or ELB/GRZ, according to the EASL recommendations, and all of them achieved an SVR (12 weeks) determined by HCV negative results by RT-PCR. The CHUC Ethics Committee approved the study (registration number CHUC-122–18). Written informed consent was provided by all the patients.

**Table 1:** Clinicopathological parameters of the patients/groups in this study.

**Study Groups**	**N**	**Sex**	**Age**	**Drugs**	**AH**	**Diab**	**Tab**	**Alc**	**Ob**	*AST/ALT	*GGT/⁎⁎**ALP**	⁎⁎**PLA**	⁎⁎**ALB**	⁎⁎**T BIL**	⁎⁎**INR**	⁎⁎⁎**Fib**	**Anti-HCV baseline**	**HCV-RNA Serum RT-PCR CET**	**HCV-RNA Serum RT-PCR baseline, SVR**		**DAAs/**	#**SVR**
**AVP**	1	Male	38	IDUs	–	–	–	–	–	-/-	-/-	+	–	–	–	F0/F1	+	+	–	3a	GP	8
	2	Male	46	IDUs	–	–	–	–	–	+/+	+/-	–	–	–	–	F0/F1	+	+	–	1a	GP	8
	3	Male	63	IDUs	–	–	–	+	–	-/-	-/-	–	–	–	–	F0/F1	+	+	–	1b	LED/SOF	8
	4	Male	57	IDUs	+	–	–	+	+	-/-	-/-	–	–	–	–	F0/F1	+	+	–	1a	LED/SOF	8
	5	Male	46	IDUs	–	–	–	–	–	-/-	-/-	–	–	–	–	F0/F1	+	+	–	4a/4c/4d	ELB/GRZ	12
	6	Male	36	IDUs	–	–	+	–	–	-/-	-/-	–	–	–	–	F0/F1	+	+	–	3a	GP	8
	7	Male	37	IDUs	–	–	–	–	–	+/+	+/+	+	+	–	+	F0/F1	+	+	–	1a	LED/SOF	8
	8	Male	37	IDUs	–	–	–	+	–	N/D/-	-/-	–	–	–	–	F0/F1	+	+	–	1a	SOF/VEL	12
	9	Male	39	IDUs	–	–	+	–	–	+/+	+/-	–	–	+	–	F0/F1	+	+	–	1a	GP	8
	10	Male	47	IDUs	–	–	–	+	+	+/+	+/-	–	–	–	–	F2	+	+	–	3a	GP	8
	11	Male	43	IDUs	–	–	–	–	–	-/-	-/-	–	–	–	–	F1	+	+	–	3a	GP	8
	12	Male	46	IDUs	–	–	–	–	–	+/+	+/-	–	–	–	–	F0/F1	+	+	–	1a	GP	8
	13	Male	39	IDUs	–	–	–	–	–	-/-	-/-	–	–	–	–	F0/F1	+	+	–	4a/4c/4d	GP	8
	14	Male	35	IDUs	–	–	–	+	–	+/+	+/-	–	–	–	–	F0/F1	+	+	–	1a	LED/SOF	12
	15	Male	43	IDUs	–	–	+	–	+	+/+	-/-	+	–	–	–	F3	+	+	–	3a	SOF/VEL	12
	16	Male	37	IDUs	–	–	–	–	–	+/+	-/-	–	–	–	+	F1	+	+	–	1a	SOF/VEL	12
	17	Male	47	IDUs	–	–	+	+	+	+/+	+/-	–	–	–	–	F4	+	+	–	4a/4c/4d	LED/SOF	8
	18	Male	55	NDUs	–	–	+	+	–	+/+	-/-	–	–	–	–	F0/F1	+	+	–	1a	LED/SOF	8
	19	Male	43	IDUs	–	–	–	–	+	+/+	-/-	–	–	–	–	F1	+	+	–	N/D	SOF/VEL	12
	20	Male	36	IDUs	–	–	+	–	–	+/+	-/-	–	–	–	–	F0/F1	+	+	–	1a	LED/SOF	8
	21	Male	39	IDUs	–	–	–	–	+	+/+	-/-	–	–	–	+	F1	+	+	–	1a	LED/SOF	8
	22	Male	34	IDUs	–	–	–	–	–	+/+	+/+	–	–	–	–	F0/F1	+	+	–	1a	LED/SOF	8
	23	Male	26	IDUs	–	–	–	–	–	+/+	+/-	–	–	–	–	F1	+	+	–	4a/4c/4d	SOF/VEL	12
	24	Male	39	NDUs	–	–	–	–	+	+/+	-/-	+	–	–	–	F0/1	+	+	–	3a	GP	8
**NAVP**	25	Male	40	NDUs	–	–	–	–	–	-/+	+/-	–	–	–	–	F0/F1	+	–	N/D	N/D	–	–
	26	Male	38	IDUs	–	–	–	–	–	+/+	+/+	–	–	–	–	F0/F1	+	–	N/D	N/D	–	–
	27	Male	47	NDUs	–	–	+	–	–	-/-	-/-	–	–	–	–	F0/F1	+	–	N/D	N/D	–	–
	28	Male	41	IDUs	–	–	+	+	+	+/+	+/-	–	–	–	–	F0/F1	+	–	N/D	N/D	–	–
	29	Male	43	IDUs	–	–	–	–	–	-/-	-/-	–	–	–	–	F0/F1	+	–	N/D	N/D	–	–
	30	Male	48	IDUs	–	–	–	–	+	N.D/N.D	N.D/N.D	–	–	–	–	F0/F1	+	–	N/D	N/D	–	–
	31	Male	40	NIDUs	–	–	–	–	–	-/+	-/-	–	–	–	–	F0/F1	+	–	N/D	N/D	–	–
	32	Male	62	NDUs	–	–	–	–	–	+/+	-/-	–	–	–	–	F0/F1	+	–	N/D	N/D	–	–
	33	Male	45	NIDUs	–	–	+	–	–	-/+	-/-	–	–	–	–	F0/F1	+	–	N/D	N/D	–	–
	34	Male	40	NIDUs	–	–	+	–	–	-/+	-/-	–	–	–	–	F0/F1	+	–	N/D	N/D	–	–
	35	Male	37	NIDUs	–	–	–	–	–	+/+	+/-	–	–	–	–	F3	+	–	N/D	N/D	–	–
	36	Male	43	NIDUs	–	–	+	–	–	-/+	-/-	–	–	–	–	F0/F1	+	–	N/D	N/D	–	–
	37	Male	34	NIDUs	–	–	–	+	–	-/+	-/-	–	–	–	–	F0/F1	+	–	N/D	N/D	–	–
**CPP**	38	Male	34	NIDUs	–	–	+	–	–	-/+	+/-	–	–	–	–	F0/F1	+	+	N/D	1a	–	–
	39	Male	57	NIDUs	+	–	+	–	–	+/+	+/-	–	–	–	–	F3	+	+	N/D	3	–	–
	40	Male	62	NIDUs	+	–	+	+	–	-/+	-/-	–	–	–	–	F0/F1	+	+	N/D	3	–	–
	41	Male	46	NIDUs	–	–	–	+	–	+/+	-/-	–	–	–	–	F2	+	+	N/D	1a	–	–
	42	Male	42	NIDUs	–	–	–	–	+	-/+	-/-	–	–	–	–	F0/F1	+	+	N/D	3	–	–
	43	Male	42	NIDUs	–	–	–	–	–	+/+	-/-	–	–	–	–	F3	+	+	N/D	3	–	–
	44	Male	40	NIDUs	–	–	–	+	+	+/+	+/+	–	–	–	–	F3	+	+	N/D	3	–	–

AVP, patients who achieved a SVR after DAAs treatment, 8–12 weeks; NAVP, patients without DAAs therapy; CPP, patients HCV positive; HCV, hepatitis C virus; IDUs, injection drug users; NIDUs, no injection drug users; NDUs, no drug users; AH, arterial hypertension; Diab, diabetes; Tab, tabaco; Alc, alcohol; Ob, obesity; T, total; Fib, fibrosis; CET, clinical evaluation time; RT-PCR, real -time PCR; PBMCs, peripheral blood mononuclear cells; RBCs, red blood cells; N/D, not determined; AST, aspartate aminotransferase; ALT, alanine aminotransferase; GTT, gamma glutamyl transpeptidase; ALP, alkaline phosphatase; PLA, platelets; ALB, albumin; BIL, bilirubin; INR, international normalized ratio; DAAs, direct-acting antivirals; GP, glecaprevir 100 mg + pibrentasvir 40 mg; LED/SOF, ledipasvir 90 mg + sofosbuvir 400 mg; SOF/VEL, sofosbuvir 400 mg + velpatasvir 100 mg; ELB/GRZ, Elbasvir 50 mg + grazoprevir 100 mg. GP and ELB/GRZ are HCV protease NS3–4a and NS5A inhibitors. LED/SOF and SOF/VEL are NS5A and NS5B inhibitors.

⁎ (-) Normal values: AST <35 U/L; ALT <45 U/L; GTT <55 U/L; (+) Altered value.

⁎⁎ Reference values: ALP 30–120 U/L; PLA 150–450 g/L; ALB 3,5–5,2 g/dL; Total BIL 0.2–1.2 mg/dL, INR 0,81–1,19; (-) Normal value; (+) Altered value.

⁎⁎⁎ Reference values Fibroscan: F0/F1: <7 kPa; F2: 7–9.5 kPa; F3: 9.6–12.5; F3/F4: 12.5–14.5 kPa; F4: >14.5 kPa; (-) Absent fibrosis; (+) Fibrosis. (-) Negative result, (+) Positive result.

# 8 or 12 weeks treatment; SVR 12 weeks; (-) No treatment/No SVR.

### 2.2 Samples collection

Blood samples of all patients/groups were collected in dry and in lithium heparin tubes. Serum was recovered from the dry tubes as previously described (Costa-Matos et al., 2013). PBMCs were isolated from the lithium heparin tubes using lymphoprep™ (Alere Technologies AS, Norway) following the manufactures instructions, and recovered PBMCs were resuspended in 200 µL of water molecular grade (G-Biosciences, USA) and also in 200 µL of roswell park memorial institute (RPMI) 1640 culture medium (Thermo Fisher Scientific, USA) supplemented with 50% fetal bovine serum (FBS) (Thermo Fisher Scientific, USA) and 20% dimethyl sulfoxide (Thermo Fisher Scientific, USA) as previously described (Silva et al., 2023). Plasma and RBCs were also collected as previously described (Silva et al., 2023). All samples were stored at −80 °C. After, serum, plasma, PBMCs resuspended in water molecular grade and RBCs resuspended in water molecular grade patients/groups samples were evaluated for the presence of HCV/OCI-RNA by ddPCR. OCI patients identification was done considering ddPCR serum negative and PBMCs positive results as previously described (Castillo et al., 2004; Silva et al., 2022; Silva et al., 2023).

### 2.3 RNA extraction, cDNA synthesis and ddPCR

Total RNA was extracted from 250 µL of serum, plasma, PBMCs resuspended in water molecular grade and RBCs resuspended in water molecular grade samples of all patients /groups using TRI Reagent LS (Sigma-Aldrich, Germany) as previously described (Silva et al., 2012; Silva et al., 2015) and using the RNeasy Mini Kit (Qiagen, Germany) following the manufactures instructions and as previously described (Silva et al., 2023). The cDNA synthesis was performed using random hexamers included in the Xpert cDNA synthesis kit (GRISP, Portugal) following the manufactures instructions as previously described (Silva et al., 2023). DdPCR was performed as previously described (Silva et al., 2023). Briefly, HCV/OCI-RNA was detected for the HCV core region using sense 5′-GCGTTAGTAYGAGTGTYG and antisense 5′-CRATTCCGGTGTACTCAC primers, and FAM-labeled HCV probe (5′-FAM-CCGCAGACCACTATGGCTC-BHQ1–3′) (Frias et al., 2019; Silva et al., 2023). The reaction mix contained 10 μL of ddPCR™ Supermix for Probes (Bio-Rad, USA), 900 nM of each primer, 250 nM of FAM-labeled HCV probe and 2 μL of template in a final volume of 22 μL. The reactions mix were placed on a Bio-Rad QX200 Droplet Digital PCR System for droplets generation following the manufactures instructions, and then HCV/OCI-RNA amplification was performed on a Bio-Rad Thermal Cycler C1000 (Bio-Rad, USA) under the cycling conditions of 95 °C for 10 min, 45 cycles at 94 °C for 30 s and 55 °C for 1 min and 98 °C for 10 min. After, the plate was read on a Bio-Rad QX200™ Droplet Reader for droplets analysis, and data was analysed using the Bio-Rad QuantaSoft™ Analysis Pro-Software v. 1.0.596 following manufactures instructions. The fluorescence amplitude threshold was automatically adjusted in individual wells. At end-point reactions the droplets are scored as positive or negative attending to the number of observed accepted droplets (10.000 or greater) and these values are used to calculate the HCV/OCI-RNA concentration using binomial Poisson statistics. Attending that ddPCR permits the absolute count of genome copies in individual wells without the requirement of an experimental or established standard curve, here, we considered the lower and the higher limits for HCV/OCI-RNA detection of 0.22 (PBMCs) and 127.34 (serum) copies/µL, respectively, as these were the lower and the higher values achieved in the analyzed blood samples.

### 2.4 Statistical analysis

Frequencies, percentages, and means were used for descriptive analysis in study group samples in Microsoft® Excel® 2016 MSO. Normal distribution was evaluated with Kolmogorov-Smirnov test. Differences in values were evaluated using Mann-Whitney test. For multiple comparisons, one-way ANOVA with Dunnett's multiple comparison test was used. Statistical analysis was performed with the SPSS (Statistics Package for Social Sciences) software version 28.0. Graphs were developed with GraphPad Prism 7.01. Significant levels were set at *p*<0.05 for all statistical analysis.

## 3 Results

### 3.1 DdPCR

OCI was presented in 18.2% of total IDUs population by ddPCR (considering serum negative and PBMCs positive results), corresponding to 20.8% in the AVP group (patients 4, 13, 15, 17 and 22) and 23.1% in the NAVP group (patients 26, 29 and 30).

**Fig. 1:**
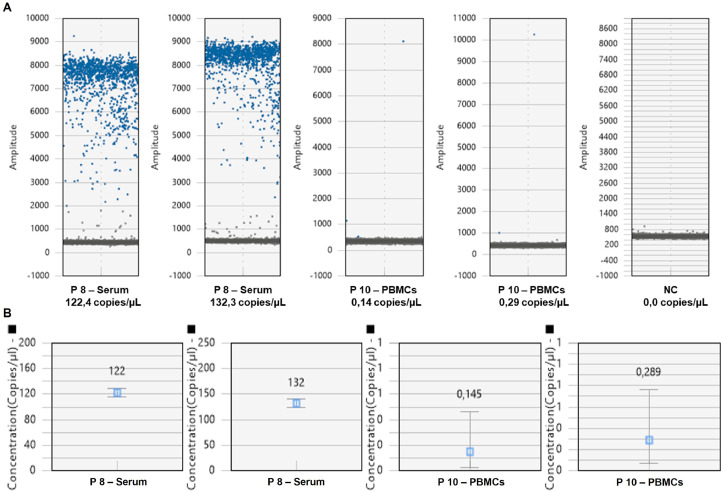
HCV/OCI-RNA detected in serum of patient 8 and in PBMCs of patient 10 in the AVP group by ddPCR, which permitted the low and the higher viral load limits detection establishment. (A) 1D fluorescence amplitude plot which shows the obtained droplets per well. (B) HCV/OCI-RNA detected in copies/µL per well. The error bars represent Poisson 95% confidence intervals.

In total analysed blood samples, HCV/OCI-RNA was detected in 65.9% of the PBMCs, 63.6% of the serum, 61.4% of the RBCs and 38.6% of the plasma samples within the lower and the higher limits of HCV/OCI-RNA detection considered for ddPCR assay in this study, that was 0.22 (PBMCs) to 127.34 (serum) copies/µL (Fig. 1 and Table 2). These values were validated by the achieved number of positive and negative droplets relatively to the obtained number of accepted droplets, as well as, by the obtained values for the binomial Poisson statistics achieved in each run (Fig. 1 and Table 2). The concentration of HCV/OCI-RNA detected in analysed blood samples and OCI patients’ identification by ddPCR are shown in Table 3. Blood samples fluorescence 1D amplitude plots are shown in Fig. S1.

**Table 2:** HCV/OCI-RNA detected in serum of patient 8 and in PBMCs of patient 10 in the AVP group by ddPCR. High (127.34 copies//µL, serum) and low (0.22, PBMCs) limit values of HCV/OCI-RNA detection were established for the ddPCR assay.

**DdPCR run**	**Sample**	**HCV/OCI-RNA (copies/µL)**	**High and low limit of detection (mean, copies/µL)**	**Poisson confidence max**	**Poisson confidence min**	**Accepted droplets**	**Positive droplets**	**Negative dropltets**	**Threshold**	**Mean amplitude of positives**	**Mean amplitude of negatives**	**Mean amplitude Total**	**Poisson confidence max**	**Poisson confidence min**
1	Serum 8	122.42	127.34	129.41	115.46	11,981	1184	10,797	1 816.72	7 310.58	439.70	1 118.70	125.98	118.86
1	Serum 8	132.26		139.67	124.89	11,596	1233	10,363	1 931.44	8 152.87	497.11	1 311.14	136.03	128.49
2	PBMCs 10	0.145	0.22	0.463	0.022	16,284	2	16,282	702.54	5 628.95	420.57	421.21	0,278	0.063
2	PBMCs 10	0.289		0.765	0.068	12,232	3	12,229	516.42	3 262.18	350.43	351.15	0,494	0.150

**Table 3:** HCV/OCI-RNA detection in blood samples of the patients/groups by ddPCR and OCI patients’ identification, considering serum negative and PBMCs positive results.

**Study groups**	**Patients**	**HCV/OCI - RNA (Copies/µL) Serum**	**Accepted droplets Serum**	**HCV/OCI - RNA (Copies/µL) Plasma**	**Accepted droplets Plasma**	**HCV/OCI - RNA (Copies/µL) PBMCs**	**Accepted droplets PBMCs**	**HCV/OCI - RNA (Copies/µL) RBCs**	**Accepted droplets RBCs**	*OCI
**AVP**	1	1.05	14,236	0.00	11,544	0.71	10,536	0.55	13,794	
	2	3.84	15,034	0.00	12,721	2.03	15,635	0.43	13,526	
	3	0.85	14,185	0.00	11,113	0.53	11,904	0.00	15,477	
	4	0.00	12,927	0.00	14,435	0.71	13,312	0.73	15,638	+
	5	0.65	14,076	0.46	11,014	0.60	11,710	0.26	15,434	
	6	3.58	14,621	0.00	12,801	0.58	16,073	0.83	15,095	
	7	4.13	11,882	0.00	12,689	1.13	14,573	0.59	13,283	
	8	127.34	11,981	0.00	14,013	1.22	15,548	0.84	11,323	
	9	1.50	13,878	0.00	11,407	1.16	15,266	0.00	13,808	
	10	0.43	16,253	1.27	13,897	0.22	12,232	0.62	17,598	
	11	1.47	13,248	0.00	12,298	0.57	11,208	0.74	18,107	
	12	0.76	13,974	0.00	15,129	0.76	14,490	0.00	14,479	
	13	0.00	13,198	0.00	13,545	0.46	11,993	0.66	17,596	+
	14	1,81	14,281	0.00	14,078	0.00	14,657	0.00	16,224	
	15	0.00	13,261	0.00	11,371	0.45	15,544	0.60	16,131	+
	16	0.00	14,122	0.00	15,279	0.00	14,528	0.24	14,647	
	17	0.00	13,289	0.00	13,217	0.71	14,943	0.37	12,659	+
	18	1.07	16,253	0.00	15,150	0.71	10,986	0.90	17,485	
	19	2.33	13,130	0.00	14,205	0.81	14,583	0.44	13,458	
	20	1.04	16,080	0.00	14,746	0.00	13,576	0.54	16,066	
	21	0.49	16,355	0.00	12,111	0.59	11,953	0.46	14,428	
	22	0.00	14,351	3.51	15,762	0.24	14,699	0.55	17,073	+
	23	0.00	11,461	0.00	12,913	0.00	17,434	0.48	14,747	
	24	0.35	16,772	0.00	14,260	0.00	14,119	1.31	17,009	
**NAVP**	25	0.67	13,999	0.00	12,834	0.43	13,680	0.00	15,494	
	26	0.00	16,334	0.00	13,082	0.68	10,321	0.47	14,345	+
	27	0.00	15,509	0.00	10,679	0.00	11,804	0.00	16,064	
	28	0.53	11,681	0.00	11,122	0.64	15,502	0.33	14,574	
	29	0.00	11,675	N/D	N/D	0.39	11,930	0.59	18,155	+
	30	0.00	11,754	0.44	13,274	0.63	11,930	0.93	16,023	+
	31	0.44	15,263	0.62	15,167	N/D	N/D	0.40	14,867	
	32	0.00	10,399	1.14	12,385	0.00	16,064	0.00	13,569	
	33	1.15	14,338	0.74	13,318	0.00	13,039	0.72	11,405	
	34	0.44	13,306	0.32	11,021	N/D	N/D	0.00	14,752	
	35	0.42	16,886	0.96	14,394	0.00	12,973	0.00	11,622	
	36	12.99	11,588	0.64	15,106	1.25	13,163	0.00	16,148	
	37	0.47	15,807	1.28	14,700	0.00	11,348	0.00	15,084	
**CPP**	38	0.28	12,687	0.00	13,223	0.00	16,550	0.00	13,408	
	43	0.96	15,737	0.43	14,671	0.00	16,026	0.00	14,659	
	40	0.61	13,490	0.74	12,161	0.00	13,240	0.00	13,892	
	41	0.00	16,499	1.92	13,632	0.25	14,823	0.00	12,159	
	42	N/D	N/D	0.63	14,861	0.25	14,172	0.00	16,790	
	43	0.00	15,770	0.37	15,395	0.46	10,129	0.00	15,828	
	44	4.84	14,547	0.61	10,601	0.68	17,433	0.37	12,674	

HCV, hepatitis C virus; OCI, occult hepatitis C infection; AVP, patients who achieved a SVR after DAAs treatment, 8–12 weeks; NAVP, patients without DAAs therapy; CPP, patients HCV positive; PBMCs, peripheral blood mononuclear cells; RBCs, red blood cells; OCI, occult hepatitis C infection; N/D, not determined.

*(+) - OCI patient.

**Fig. 2:**
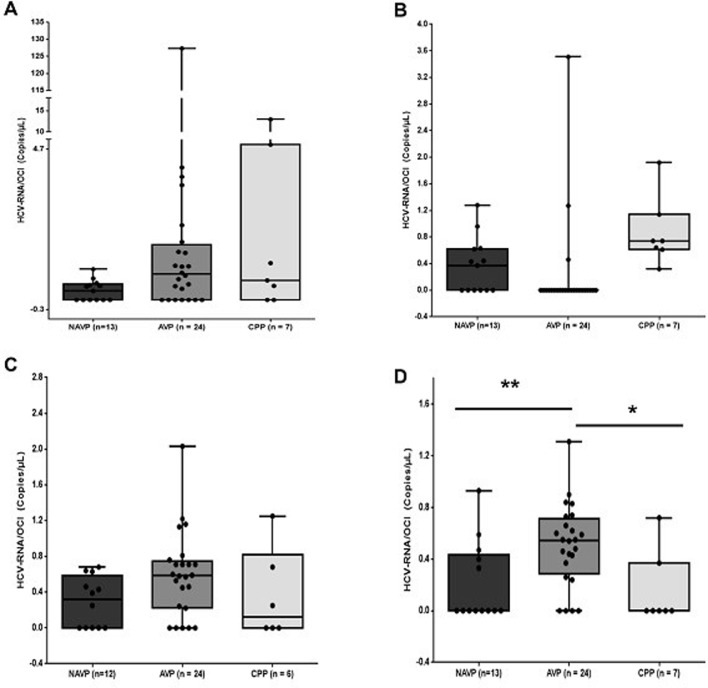
HCV/OCI-RNA detected in the blood samples of the patients in the AVP, NAVP and CPP groups. (A) Serum, (B) Plasma, (C) PBMCs and (D) RBCs. In total, higher values statistically significant of HCV/OCI-RNA were achieved in RBCs samples of the patients in the AVP group comparatively to NAVP (*p*<0.01) and CPP (*p* < 0.05) patients’ groups by ddPCR, although for the other blood samples no statistically significant differences were shown.

Within each group, 83.3% of HCV/OCI-RNA was detected in RBCs, 79.2% in PBMCs, 70.8% in serum and 12.5% in plasma samples of the patients in the AVP group. In patients of the NAVP group 53.9% was detected in serum, plasma and PBMCs and 38.5% in RBCs samples. In patients of the CPP group, 100% was detected in plasma, 57.1% in serum, 42.9% in PBMCs and 28.6% in RBCs samples. In total, higher values statistically significant of HCV/OCI-RNA were detected in RBCs samples of the patients in the AVP group comparatively to the NAVP (*p*<0.01) and CPP (*p* < 0.05) patients’ groups by ddPCR, although for the other blood samples no statistically significant differences were shown (Fig. 2).

## 4 Discussion

OCI is characterized by the detection of HCV/OCI in PBMCs samples of the patients without detection in serum samples by conventional PCR assays (Castillo et al., 2004; Austria and Wu, 2018; Wroblewska et al., 2021). We have recently performed two studies in OCI where we have characterized OCI patients, considering serum negative and PBMCs positive results by ddPCR, in different study groups and also their possibility of HCV/OCI transmission (Silva et al., 2022; Silva et al., 2023). The OCI was identified in HCV negative patients (CNP group), patients who spontaneously eliminate HCV (SEP group) and in patients who achieved a SVR after DAAs therapy (AVP group) (Silva et al., 2022). Here, we evaluate the presence of OCI in IDUs, NIDUs and NDUs patients in the AVP and NAVP groups by ddPCR, considering also serum negative and PBMCs positive results. Additionally, plasma and RBCs samples of the patients in both groups were also screened for HCV/OCI-RNA detection by ddPCR.

OCI was detected in 18.2% IDUs seropositive patients in the AVP (*n* = 24) and NAVP (*n* = 13) groups by ddPCR. We have not found other studies reporting the percentage of HCV/OCI-RNA detection by ddPCR, while a study reporting HCV detection in HIV-coinfected patients with long-term SVR using ddPCR was previously described (Frias et al., 2019). Although, studies reporting OCI of 9.6% (*n* = 11) and 23.6% (*n* = 174) in seronegative and/or seropositive IDUs and in patients with chronic hepatitis C/healthy individuals, respectively, by reverse transcription PCR were previously published (Sheikh et al., 2019; Abd Alla et al., 2017). Moreover, OCI of 3.9% (*n* = 1280), 11.3% (*n* = 150), 12.9% (*n* = 70), 20% (*n* = 100) and 8.1% (*n* = 11) identified in HCV patients who achieved SVR (12 or 24 weeks), by reverse transcription PCR or RT-PCR, were also previously described (Hedayati-Moghaddam et al., 2021; Yousif et al., 2018; Mekky et al., 2019; Mashaal et al., 2022; Khattab et al., 2023).

Taking in consideration our results we suggest that the patients in the AVP group might not be entirely cured, and studies reporting treatment failures after GP, LED/SOF, SOF/VEL and ELB/GRZ were previously described (Attar and Van Thiel, 2015; Lybeck et al., 2019; Ghany et al., 2020; Zahid et al., 2022). Our findings questioning the higher DAAs effectiveness described in the literature, that carefully deserves investigation about the meaning of this discrepancy and related clinical significance.

Considering the patients in the NAVP group, obtained results suggest that tested patients at clinical evaluation time were not identified as HCV positive by RT-PCR, probably as just serum samples were screened at that time. These results efforts attention in the currently serum samples screening for HCV diagnose, and also efforts the OCI definition when the screening of PBMCs samples for HCV/OCI-RNA detection was considered.

Furthermore, in total HCV/OCI-RNA was detected in 70.8%, 12.5%, 79.2% and 83.3% of the serum, plasma, PBMCs and RBCs samples, respectively. Higher values, statistically significant, of HCV/OCI-RNA were achieved in RBCs samples of the patients in the AVP, comparatively to patients in the NAVP (*p*<0.01) and CPP (*p* < 0.05) groups, by ddPCR, and the detection of HCV in RBCs was previously described (Schmidt et al., 1997; Lotz et al., 2002; Simon et al., 2003). These results suggest that RBCs, as PBMCs (Hanno et al., 2014), could be a predictor for HCV/OCI identification in future. Moreover, overall results shown also that ddPCR could be a more sensitive technology for HCV/OCI-RNA detection, as a range of 0.22 to 127.34 copies/µL were able to be detected.

In conclusion, OCI was identified in IDUs patients in the AVP and NAVP groups by ddPCR, suggesting that OCI patients in the AVP group might not have a total viral eradication, and that OCI patients in the NAVP group were not identified at clinical evaluation time due probably as just serum samples were tested at that time. HCV-RNA was also detected in no OCI patients in the AVP and NAVP groups. Overall results suggest that the HCV/OCI identification in patients with sustained viral response after DAAs therapy and those who spontaneously cleared the virus should be performed more accurately in future, as well as, in the diagnose of patients suspected of being infected with the virus. Additionally, PBMCs and RBCs samples are suggested as predictors for HCV/OCI diagnosis and management in future preventing HCV/OCI transmission. The epidemiological and clinical meaning of this findings deserves further investigation.

## Funding

This work was supported by the 10.13039/501100001871Foundation for Science and Technology (FCT) under the grant number PTDC/SAU-SER/30,788/2017, FEDER.

## Author contributions

Eliane Silva and Armando Carvalho designed the study. Armando Carvalho, Adélia Simão, João Madaleno and Bernardo Canhão participated in the patient's recruitment, data collection, and data interpretation and all together with Eliane Silva participated in the sample's collection. Eliane Silva performed the ddPCR at the Cancer Biology & Epigenetics Group, Research Center of IPO Porto. Sara Marques and Bárbara Leal performed the statistical analysis. Eliane Silva wrote the paper with Sara Marques support. All authors critically reviewed the manuscript and approved the final version of the manuscript for publication.

## Declaration of Competing Interest

The authors declare that they have no known competing financial interests or personal relationships that could have appeared to influence the work reported in this paper.

## Data Availability

Data will be made available on request.

## Appendix Supplementary materials

Image, application 1

## References

[bib0001] Castillo I., Pardo M., Bartolome J., Ortiz-Movilla N., Rodriguez-Inigo E., de Lucas S. (2004). Occult hepatitis C virus infection in patients in whom the etiology of persistently abnormal results of liver-function tests is unknown. J. Infect. Dis..

[bib0002] Austria A., Wu G.Y. (2018). Occult Hepatitis C virus infection: a review. J. Clin. Transl. Hepatol..

[bib0003] Hedayati-Moghaddam M.R., Soltanian H., Ahmadi-Ghezeldasht S. (2021). Occult hepatitis C virus infection in the middle east and eastern mediterranean countries: a systematic review and meta-analysis. World J. Hepatol..

[bib0004] Naghdi R., Ranjbar M., Bokharaei-Salim F., Keyvani H., Savaj S., Ossareh S. (2017). Occult Hepatitis C infection among hemodialysis patients: a prevalence study. Ann. Hepatol..

[bib0005] Frias M., Rivero-Juarez A., Tellez F., Palacios R., Jimenez-Arranz A., Pineda J.A. (2019). Evaluation of hepatitis C viral RNA persistence in HIV-infected patients with long-term sustained virological response by droplet digital. PCR. Sci. Rep..

[bib0006] Wroblewska A., Bielawski K.P., Sikorska K. (2021). Occult infection with hepatitis C virus: looking for clear-cut boundaries and methodological consensus. J. Clin. Med..

[bib0007] Yousif M.M., Elsadek Fakhr A., Morad E.A., Kelani H., Hamed E.F., Elsadek H.M. (2018). Prevalence of occult hepatitis C virus infection in patients who achieved sustained virologic response to direct-acting antiviral agents. Infez. Med..

[bib0008] Wang Y., Rao H., Chi X., Li B., Liu H., Wu L. (2019). Detection of residual HCV-RNA in patients who have achieved sustained virological response is associated with persistent histological abnormality. EBioMedicine.

[bib0009] Martinez-Rodriguez M.L., Uribe-Noguez L.A., Arroyo-Anduiza C.I., Mata-Marin J.A., Benitez-Arvizu G., Portillo-Lopez M.L. (2018). Prevalence and risk factors of Occult Hepatitis C infections in blood donors from Mexico City. PLoS ONE.

[bib0010] Helaly G.F., Elsheredy A.G., El Basset Mousa A.A., Ahmed H.K.F., Oluyemi A.E.S (2017). Seronegative and occult hepatitis C virus infections in patients with hematological disorders. Arch. Virol..

[bib0011] Sheikh M., Bokharaei-Salim F., Monavari S.H., Ataei-Pirkooh A., Esghaei M., Moradi N. (2019). Molecular diagnosis of occult hepatitis C virus infection in Iranian injection drug users. Arch. Virol..

[bib0012] Donyavi T., Bokharaei-Salim F., Khanaliha K., Sheikh M., Bastani M.N., Moradi N. (2019). High prevalence of occult hepatitis C virus infection in injection drug users with HIV infection. Arch. Virol..

[bib0013] Sugden P.B., Pham T.N., Ratnarajah S., Cameron B., Bull R., White P.A. (2013). Rare occurrence of occult hepatitis C virus in apparently uninfected injecting drug users: a two-centre, masked, case-control study. J. Viral Hepat..

[bib0014] Enkelmann J., Gassowski M., Nielsen S., Wenz B., Ross S., Marcus U. (2020). High prevalence of hepatitis C virus infection and low level of awareness among people who recently started injecting drugs in a cross-sectional study in Germany, 2011-2014: missed opportunities for hepatitis C testing. Harm. Reduct. J.

[bib0015] Scheinmann R., Hagan H., Lelutiu-Weinberger C., Stern R., Des Jarlais D.C., Flom P.L. (2007). Non-injection drug use and Hepatitis C Virus: a systematic review. Drug Alcohol Depend..

[bib0016] Van den Berg C.H., van de Laar T.J., Kok A., Zuure F.R., Coutinho R.A., Prins M. (2009). Never injected, but hepatitis C virus-infected: a study among self-declared never-injecting drug users from the Amsterdam Cohort Studies. J. Viral Hepat..

[bib0017] Schuch-Goi S.B., Scherer J.N., Kessler F.H.P., Sordi A.O., Pechansky F., von Diemen L. (2017). Hepatitis C: clinical and biological features related to different forms of cocaine use. Trends Psychiatry Psychother.

[bib0018] Elmasry S., Wadhwa S., Bang B.R., Cook L., Chopra S., Kanel G. (2017). Detection of occult hepatitis C virus infection in patients who achieved a sustained virologic response to direct-acting antiviral agents for recurrent infection after liver transplantation. Gastroenterology.

[bib0019] Mohamed A.A., Eljaky A.M., Abdelsameea E.M., Fouad T.R., El-Ezawy H.E.-.D.M (2019). Prevalence and effect of occult hepatitis C infection in patients with persistent liver enzyme elevation after achieving 24 weeks of sustained virological response. Egypt. J. Intern. Med..

[bib0020] Mekky M.A., Sayed H.I., Abdelmalek M.O., Saleh M.A., Osman O.A., Osman H.A. (2019). Prevalence and predictors of occult hepatitis C virus infection among Egyptian patients who achieved sustained virologic response to sofosbuvir/daclatasvir therapy: a multi-center study. Infect. Drug Resist.

[bib0021] Attar B.M., Van Thiel D. (2015). A New Twist to a chronic HCV infection: occult Hepatitis C. Gastroenterol. Res. Pract..

[bib0022] Lybeck C., Brenndorfer E.D., Sallberg M., Montgomery S.M., Aleman S., Duberg A.S. (2019). Long-term follow-up after cure from chronic hepatitis C virus infection shows occult hepatitis and a risk of hepatocellular carcinoma in noncirrhotic patients. Eur. J. Gastroenterol. Hepatol..

[bib0023] Ghany M.G., Morgan T.R., Panel A-IHCG (2020). Hepatitis C guidance 2019 update: American Association for the study of liver diseases-infectious diseases society of america recommendations for testing, managing, and treating hepatitis C virus infection. Hepatology.

[bib0024] Zahid H., Aslam K., Dahl E.H., Abbassi W., Adan S., Van den Bergh R. (2022). DAA treatment failures in a low-resource setting with a high burden of hepatitis C infections: a case series. Oxf. Med. Case Rep..

[bib0025] Silva E., Marques S., Salta S., Sequeira J.P., Madaleno J., Simão A., Carvalho A. (2022). Occult hepatitis C infection detection in people who use drugs with or without direct-antiviral agents therapy. Int. Liver Congress 2022; London, United Kingdom: J. Hepatol..

[bib0026] Silva E., Marques S.;., Osorio H., Canhão B., Madelo J., Simão A. (2023). Occult hepatitis C infection: viruses with infectious potential in Huh7.5 and MDBK cell lines suggest HCV/OCI transmission. Pharm. Pharmacol. Int. J..

[bib0027] Costa-Matos L., Batista P., Monteiro N., Henriques P., Girao F., Carvalho A. (2013). Hfe mutations and iron overload in patients with alcoholic liver disease. Arq. Gastroenterol..

[bib0028] Silva E., Marques S., Osorio H., Carvalheira J., Thompson G (2012). Endogenous hepatitis C virus homolog fragments in European rabbit and hare genomes replicate in cell culture. PLoS ONE.

[bib0029] Silva E., Osorio H., Thompson G. (2015). Hepatitis C-like viruses are produced in cells from rabbit and hare DNA. Sci. Rep..

[bib0030] Abd Alla M.D.A., Elibiary S.A., Wu G.Y., El-Awady M.K (2017). Occult HCV Infection (OCI) diagnosis in cirrhotic and non-cirrhotic naive patients by Intra-PBMC Nested Viral RNA PCR. J. Clin. Transl. Hepatol..

[bib0031] Mashaal A.R., Abd El-Hameed M., El Ray A.A., Mahmoud Diab T., Dawood R.M., Shemis M.A. (2022). Detection of occult Hepatitis C virus infection in egyptian patients who achieved a sustained virologic response to direct-acting antiviral agents. Asian Pac. J. Cancer Prev..

[bib0032] Khattab M.A., Zakaria Y., Sadek E., Abd El Fatah A.S., Fouad M., Khattab M. (2023). Detection of hepatitis C virus (HCV) RNA in the peripheral blood mononuclear cells of HCV-infected patients following sustained virologic response. Clin. Exp. Med..

[bib0033] Schmidt W.N., Wu P., Han J.Q., Perino M.J., LaBrecque D.R., Stapleton J.T. (1997). Distribution of hepatitis C virus (HCV) RNA in whole blood and blood cell fractions: plasma HCV RNA analysis underestimates circulating virus load. J. Infect. Dis..

[bib0034] Lotz G., Szalay F., Firneisz G., Abonyi M., Lengyel G., Telegdy L. (2002). Localization of hepatitis C virus RNA on human erythrocytes by RT in situ PCR technique. Scand. J. Gastroenterol..

[bib0035] Simon S., Lotz G., Kury F., Reipert B., Steinkasserer A., Eibl M.M., Schinazi RF, Sommadossi J-P, Rice CM (2003). Frontiers in Viral Hepatitis.

[bib0036] Hanno A.F.F., Mohiedeen K.M., Alshayeb A.F., Deghedy A. (2014). HCV RNA in peripheral blood mononuclear cells (PBMCs) as a predictor of the response to antiviral therapy in chronic hepatitis C. Alexandria J. Med..

