# Supplementary material for: Occult hepatitis C infection identified in injection drug users with direct antiviral agents therapy and spontaneous resolution of hepatitis C virus infection
Source: Virus Res. 2023 Apr 2;329:199104. doi: 10.1016/j.virusres.2023.199104 (PMC10194197; doi:10.1016/j.virusres.2023.199104)
Supplement: Supplementary file 1 [file mmc1.docx]

**Supplementary Materials**

**Supplementary Fig. S1**

**Fig. S1** Fluorescence 1D amplitude plots obtained for the analysed blood samples of the patients by ddPCR under the established low and higher limit of HCV/OCI-RNA detection. (A, B, C, D, E and F) Fluorescence 1D amplitude plots obtained for analysed serum (S) samples of the patients/groups. (G, H, I, J and K) Fluorescence 1D amplitude plots obtained for analysed PBMCs samples (PB) of the patients/groups. (L, M, N and O) Fluorescence 1D amplitude plots obtained for analysed RBCs (RB) samples of the patients/groups. (P, Q and R) Fluorescence 1D amplitude plots obtained for analysed plasma (P) samples of the patients/groups.
